# Development of central retinal artery occlusion accompanied by choroidal folds in a patient with antineutrophil cytoplasmic antibody-associated vasculitis: A case report

**DOI:** 10.1097/MD.0000000000021934

**Published:** 2020-08-28

**Authors:** Mai Takagi, Takatoshi Kobayashi, Teruyo Kida, Nanae Takai, Hiromi Shoda, Koichi Maruyama, Rei Tada, Shigeki Makino, Tsunehiko Ikeda

**Affiliations:** a Department of Ophthalmology, Osaka Medical College; b Maruyama Eye Clinic, Takatsuki; c Tada Eye Clinic, Ikeda; d Department of Internal Medicine, Osaka Medical College, Takatsuki-City, Osaka, Japan.

**Keywords:** antineutrophil cytoplasmic antibody, central retinal artery occlusion, myeloperoxidase, optical coherence tomography

## Abstract

**Rationale:**

We report a case of central retinal artery occlusion (CRAO) accompanied by choroidal folds in a patient positive for myeloperoxidase (MPO)-antineutrophil cytoplasmic antibody (ANCA).

**Patient concerns:**

The study involved a 67-year-old female patient who presented at the Department of Ophthalmology, Osaka Medical College, Takatsuki-City, Osaka, Japan on October 24, 2016 after becoming aware of a sudden decrease of visual acuity (VA) in her right eye. Other than suffering with scleritis 6-months previous, there was no obvious past history.

**Diagnosis:**

Upon examination, the VA in her right eye was hand motion, and the anterior segment of that eye showed thinning of the superior sclera. Macular edema in the inner retina and cherry red spots were observed in the ocular fundus, and optical coherence tomography (OCT) findings showed hyperreflectivity of the inner retina and choroidal folds. Fluorescein angiography (FA) examination of the fundus showed scattered areas of no retinal perfusion, and indocyanine green angiography (IA) findings of the fundus indicated a possible choroidal circulatory disturbance in her right eye. Blood test findings revealed the patient to be positive for MPO-ANCA. Based on the above findings, the patient was diagnosed with CRAO and choroidal circulatory disturbance due to ANCA-associated vasculitis.

**Interventions:**

For treatment, steroid semi-pulse therapy was initiated.

**Outcomes:**

Post treatment initiation, the fundus features and choroidal folds gradually improved, and her VA slightly improved to 0.08.

**Lessons:**

Based on the FA, IA, and OCT findings, the present case was considered to have CRAO accompanied by choroidal circulatory disturbance due to ANCA-associated vasculitis, a rare disease that may be complicated by choroidal circulatory disturbances.

## 1 Introduction

Antineutrophil cytoplasmic antibody (ANCA), an immunoglobulin G autoantibody against human neutrophil cytoplasm, is known to cause various vasculitis-associated symptoms. Vasculitis is broadly classified as systemic or localized vasculitis, with ANCA-associated systemic vasculitis being classified into 3 types:

1. microscopic polyangiitis (MPA),
2. eosinophilic granulomatosis with polyangiitis (EGPA), and
3. granulomatosis with polyangiitis (GPA).^[1]^

Patients with MPA or EGPA are often found positive for myeloperoxidase (MPO)-ANCA, and those with GPA are often found positive for proteinase 3 (PR3)-ANCA.

While ANCA-associated vasculitis causes various eye symptoms, mainly proptosis, scleritis, episcleritis, conjunctivitis, blepharitis, keratitis, uveitis, and retinal vasculitis,^[2,3]^ central retinal artery occlusion (CRAO) has also been reported.^[4–27]^ Here, we describe a MPO-ANCA-positive patient who developed CRAO accompanied by choroidal folds. The findings in this present case suggest that circulatory disturbance of not only the retina, but also the choroid, might be involved in the pathology of CRAO.

## 2 Case report

In this study, we report the case of a 67-year-old female who presented after becoming aware of a sudden decrease of visual acuity (VA) in her right eye. In April 2016, hyperemia was noted in the patient's right eye, and in July 2016, the patient was diagnosed with scleritis. Symptoms were relieved after the patient was treated with predonine 30 mg and steroid eye drops. In mid October 2016, the patient experienced general malaise and fever, and subsequently presented at the Department of Internal Medicine at a nearby hospital. Upon examination, inflammatory responses were observed, as indicated by a C-reactive protein (CRP) level of 33 mg/dL and a white blood cell (WBC) count of 26,000/μL. On October 24, 2016, the patient became aware of a sudden decrease of VA in her right eye and presented at the Department of Ophthalmology, Osaka Medical College, Takatsuki-City, Osaka, Japan for a detailed examination. This case study was approved by the Ethics Committee of Osaka Medical College, and was performed in accordance with the tenets set forth in the Declaration of Helsinki. Informed written consent was obtained from the patient for publication of this case report and the accompanying images.

**Figure 1:**
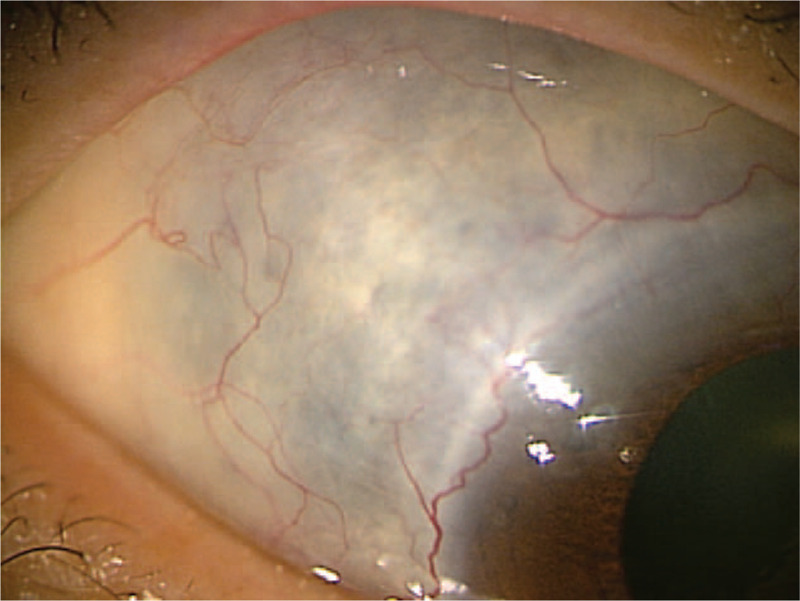
Slit-lamp microscopy image of the patient's right eye obtained at the initial presentation showing no obvious inflammation in the anterior chamber, yet thinning of the sclera on the superior temporal aspect.

**Figure 2:**
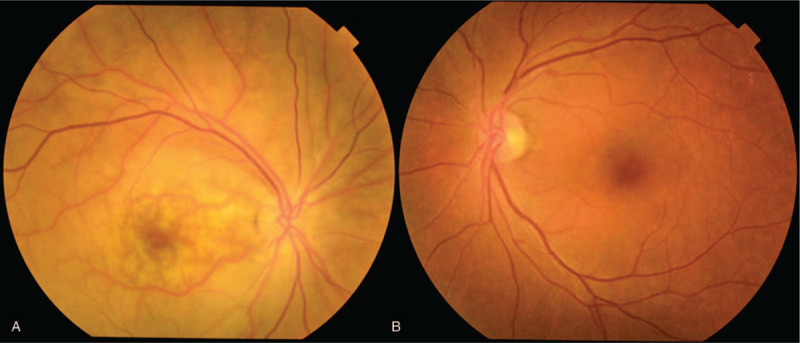
Fundus images of the patient's right eye (A) and left eye (B) obtained at the initial presentation showing cherry-red-color spots in the macular area of the right eye, yet no obvious abnormalities in the left eye.

**Figure 3:**
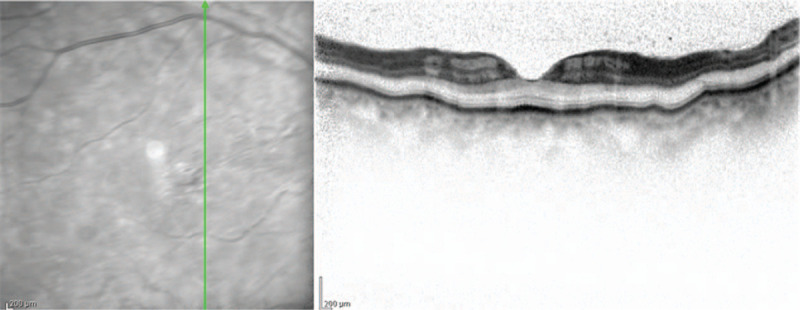
OCT image of the patient's right eye obtained at the initial presentation showing hyperreflectivity and edema in the inner retina, as well as an irregular ellipsoid zone and choroidal folds.

**Figure 4:**
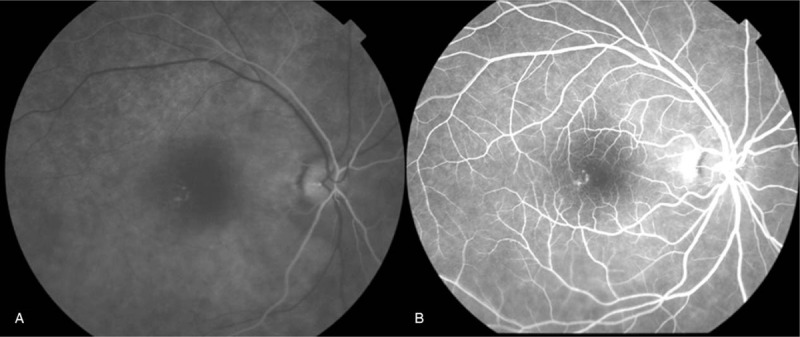
Fluorescein angiography (FA) images of the patient's right eye (A) and left eye (B) obtained at the initial presentation. A delayed arm-to-retinal circulation time of 18 s and scattered areas of no retinal perfusion was observed in the periphery of the right eye, however, no obvious abnormality was observed in the left eye.

**Figure 5:**
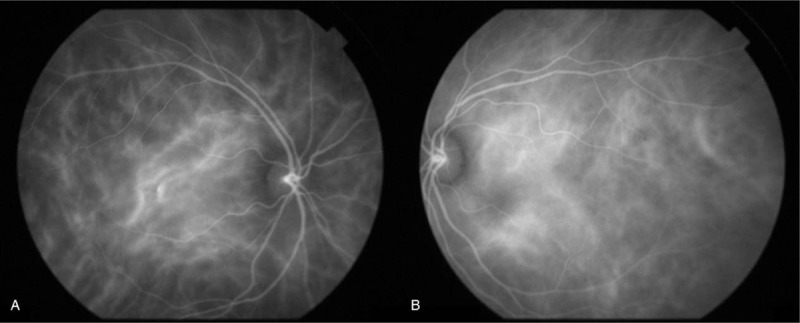
Indocyanine green angiography (IA) images of the patient's right eye (A) and left eye (B) obtained at the initial presentation. Overall, the right eye appeared slightly more hypofluorescent than the left eye.

Upon examination, the patient's corrected VA was hand motion at 50 cm in her right eye and 1.2 in her left eye. Intraocular pressure was 5 mm Hg in her right eye and 9 mm Hg in her left eye. The light reflex was decreased in the right eye, which was positive for relative afferent pupillary defect. Slit-lamp microscopy examination revealed no obvious inflammation in the anterior chamber, yet did reveal thinning of the sclera on the superior temporal aspect (Fig. 1). Fundus examination revealed retinal edema with a macular cherry-red spot appearance in her right eye (Fig. 2). At her initial visit, an optical coherence tomography (OCT) examination revealed hyperreflectivity and edema in the inner retina, as well as an irregular ellipsoid zone and choroidal folds, in her right eye (Fig. 3). No abnormalities were observed in the left eye. Fluorescein angiography (FA) of the fundus showed a delayed arm-to-retinal circulation time of 18 s and scattered areas of no retinal perfusion in the periphery (Fig. 4). Although indocyanine green angiography (IA) of the fundus showed no delay in choroidal circulation time, the right eye, overall, appeared slightly more hypofluorescent than the left eye (Fig. 5), and pale areas of excessive leakage were observed in the late phase.

Blood test results were as follows: WBC count, 16.4 × 10^3^/μL; erythrocyte sedimentation rate, 61 mm/h; CRP, 18.11 mg/dL; ferritin, 1839.0 ng/mL: MPO-ANCA, 70.3 U/mL (reference value: <3.5 U/mL); PR3-ANCA, <1.0 U/mL, and a D-dimer, 9.5 μg/mL (reference value: <5 ng/mL). There were no particular abnormalities in the skin or neurologic findings. Since a magnetic resonance imaging examination of the patient's head showed a beaded dilatation of the temporal artery, a temporal arterial biopsy was performed. Although infiltration of lymphocytes and plasma cells was observed, no giant cells were detected. Thus, there was no diagnosis of temporal arteritis. Based on these findings, the patient was diagnosed as CRAO due to ANCA-associated vasculitis, and steroid semi-pulse therapy was started.

After steroid semi-pulse therapy was administered, the systemic condition was markedly improved, and predonine was tapered. On November 22, 2016, a combined therapy of predonine (50 mg/day) and azathioprine (50 mg/day) was initiated. On December 8, 2016, an examination revealed that the patient had become negative for MPO-ANCA. Retinal edema with a macular cherry-red spot appearance in her right eye resolved, and VA was improved to 0.08. An OCT examination performed 1 month after the initiation of treatment showed thinning of the inner retina, however, the choroidal folds were found to have completely disappeared.

## 3 Discussion

Types of ANCA include perinuclear-ANCA (p-ANCA), which is characterized by staining of the perinuclear cytoplasm, and cytoplasmic-ANCA (c-ANCA), which is characterized by staining of the whole cytoplasm. The typical antigens for p-ANCA and c-ANCA are MPO and PR3, respectively. It has been reported that positivity for p-ANCA is common in MPA, and that positivity for c-ANCA is common in GPA.^[1]^

There have recently been increasing reports of ocular complications associated with ANCA-associated vasculitis.^[2,3]^ GPA (a typical type of c-ANCA-associated vasculitis), which had been referred to as Wegener's granulomatosis, is reportedly associated with ocular complications including scleritis, orbital inflammatory pseudotumor, peripheral corneal ulcer, iritis, and ocular ischemia due to temporal arteritis; and there are also reports of concurrent onset of CRAO.^[5–15]^ For MPA (a type of p-ANCA-associated vasculitis), the reported ocular complications include conjunctival lithiasis, peripheral corneal ulcer, and retinal vein occlusion. For EGPA, which had been referred to as allergic granulomatous angiitis and Churg-Strauss syndrome, uveitis, retinal artery occlusion, and other ocular complications are reported. A number of studies have shown that CRAO due to ANCA-associated vasculitis concurrently occurred with EGPA.^[16–28]^ The Birmingham Vasculitis Activity Score, an indicator for the assessment of ANCA-associated vasculitis activity, includes decreased VA, which is a symptom of CRAO.^[29]^ Thus, CRAO is regarded as an important comorbid eye symptom in patients with ANCA-associated vasculitis.

In previous studies, it has been considered that EGPA is attributed to hypercoagulability due to eosinophils,^[4,13]^ and that it commonly occurs in people aged 50 years or older. Thus, aging and the development of intravascular plaque might predispose people to CRAO. Our patient was also relatively old (67 years). Although EGPA was not diagnosed, she was found positive for MPO-ANCA, which is considered to be rather specifically detected in EGPA. Thus, she may have been in a state of hypercoagulability. Furthermore, EGPA histopathologically causes eosinophilic infiltration and extravascular granuloma around small vessels (mainly arterioles). In our patient, the FA findings showed scattered segments of occluded retinal capillaries in the mid-periphery in the arteriovenous phase. This suggests that CRAO might have occurred in the following process. First, in the early stage of onset, there was vasculitis at the arteriolar and venular levels, and capillary occlusion. As time progressed, inflammation spread from the capillaries to small- and medium-sized vessels, thus causing inflammation and occlusion of the central retinal artery, which might have ultimately led to CRAO. Hence, for the treatment of CRAO due to ANCA-associated vasculitis, steroid therapy for the primary disease is basically the first-line therapy.^[30]^

In our patient, the possible characteristic findings were the hyperreflectivity of the inner retina on the OCT scans and the presence of choroidal folds at her initial visit. Since the cherry red spots were somewhat atypical, our patient might have had an incomplete form of CRAO. However, previous reports have indicated that vasculitis may affect not only retinal but also choroidal circulation as it spreads to the ophthalmic artery and smaller vessels.^[28,31,32]^ In our patient, the IA findings revealed circulatory disturbance mainly in the small vessels of the choroid in the early phase, and fluorescence leakage from the choroidal vessels in the late phase. This suggests that vasculitis might have affected not only the retina, but also the choroid. While choroidal folds can be induced by various causes, Bullock et al reported that they might be the result of congestion of choroidal circulation and inflammation of choroidal vessels, in addition to ocular compression due to tumors, ocular hypotension, and scleritis.^[33]^ In our patient, choroidal folds appear to have been induced by hypercoagulability or inflammation of choroidal vessels due to ANCA-associated vasculitis. This is also fully plausible, because the choroidal folds in our patient completely disappeared after the administration of the steroid semi-pulse therapy.

When ANCA-associated vasculitis is complicated by eye symptoms, therapeutic intervention with steroids is generally considered necessary.^[30]^ Although the prognosis of CRAO due to ANCA-associated vasculitis is generally considered to be poor, early therapeutic intervention may result in the recovery of a relatively favorable VA.^[18]^ In our patient, the improvement of VA might have been due to the fact that steroid semi-pulse therapy was also initiated soon after she became aware of the sudden decreased of VA. Hence, and as our findings indicate, in patients with CRAO associated with general symptoms, it is important to perform detailed physical examinations, including a blood test, as needed, in order to identify the cause, and to start treatment early, while keeping the possibility of conditions secondary to ANCA-associated vasculitis in mind.

## Acknowledgments

The authors wish to thank John Bush for editing the manuscript.

## Author contributions

**Conceptualization:** Mai Takagi, Takatoshi Kobayashi, Tsunehiko Ikeda.

**Data curation:** Mai Takagi, Takatoshi Kobayashi, Teruyo Kida, Shigeki Makino, Tsunehiko Ikeda.

**Formal analysis:** Mai Takagi, Takatoshi Kobayashi, Tsunehiko Ikeda.

**Funding acquisition:** Mai Takagi, Takatoshi Kobayashi, Tsunehiko Ikeda.

**Investigation:** Mai Takagi, Takatoshi Kobayashi, Teruyo Kida, Nanae Takai, Hiromi Shoda, Koichi Maruyama, Rei Tada, Shigeki Makino, Tsunehiko Ikeda.

**Methodology:** Mai Takagi, Takatoshi Kobayashi, Teruyo Kida, Nanae Takai, Hiromi Shoda, Shigeki Makino, Tsunehiko Ikeda.

**Resources:** Mai Takagi.

**Supervision:** Teruyo Kida, Nanae Takai, Koichi Maruyama, Rei Tada, Shigeki Makino, Tsunehiko Ikeda.

**Validation:** Mai Takagi, Tsunehiko Ikeda.

**Visualization:** Mai Takagi, Tsunehiko Ikeda.

**Writing – original draft:** Mai Takagi, Takatoshi Kobayashi, Tsunehiko Ikeda.

**Writing – review & editing:** Teruyo Kida, Nanae Takai, Hiromi Shoda, Koichi Maruyama, Rei Tada, Shigeki Makino.
